# Umfrage zur hausärztlichen Versorgungsrealität bei rheumatologischen Patient:innen in Unterfranken und zum Beitrag der Digitalisierung

**DOI:** 10.1007/s00393-025-01754-5

**Published:** 2025-12-01

**Authors:** Patrick-Pascal Strunz, Michael Gernert, Lea-Kristin Nagler, Hannah Labinsky, Matthias Fröhlich, Maurice Stetter, Clara Stetter, Lotte Possler, Marc Schmalzing

**Affiliations:** 1 Medizinische Klinik II, Rheumatologie/Immunologie, Universitätsklinikum Würzburg, Würzburg, Oberdürrbacher Straße 6, 97080 Würzburg, Deutschland; 2 Sanitätszentrum Veitshöchheim, Sanitätsdienst der Bundeswehr, Veitshöchheim, Deutschland; 3 Abteilung für Innere Medizin, Main-Klinik Ochsenfurt, Ochsenfurt, Deutschland

**Keywords:** Grundversorgung, Rheumatische Erkrankungen, Hausärzt:innen, Digitale Gesundheitsanwendung (DiGA), Ländliche Versorgung, Primary care, Rheumatic diseases, General practitioners, Digital health application (DHA), Rural healthcare

## Abstract

**Hintergrund:**

Durch den demografischen Wandel wird sich die bestehende rheumatologische Mangelversorgung in Deutschland vor allem im ländlichen Raum weiter verschärfen. Als Ansätze zur Verbesserung der Versorgung rheumatologischer Patient:innen werden daher eine vermehrte Einbindung von Hausärzt:innen und mehr Digitalisierung vorgeschlagen.

**Ziel der Arbeit:**

Erhebung des Status quo der hausärztlichen Versorgung rheumatologischer Patient:innen unter besonderer Berücksichtigung des Einsatzes von Digitalisierung.

**Material und Methoden:**

Zur Beantwortung dieser Fragen erfolgte eine Querschnittsstudie unter Hausärzt:innen im Bereich des Rheumazentrums Würzburg unter Verwendung eines Fragebogens mit 10 Domänen und 13 Fragenitems.

**Ergebnisse:**

An der Umfrage nahmen 92 Hausärzt:innen teil, was etwa 10 % der in Unterfranken tätigen Hausärzt:innen entspricht. Im Schnitt werden 3,9 Patient:innen mit Verdacht auf eine rheumatische Erkrankung pro Quartal gesehen und 22 rheumatologische Patient:innen mitbetreut. 62 % trauen sich eine eigenverantwortliche Versorgung von stabil eingestellten rheumatologischen Patient:innen zu. Hierfür bezieht der Großteil (94,6 %) seine Informationen bereits jetzt aus digitalen Quellen. Nur 30,4 % kennen digitale Fortbildungen und nur 3 % nutzen Screeningtools auf entzündlich-rheumatische Erkrankungen. DiGAs wurden bereits von 61 % verordnet.

**Schlussfolgerung:**

Hausärzt:innen tragen bereits jetzt erheblich zur Versorgung rheumatologischer Patient:innen bei und wären auch bereit dazu, stabil eingestellte rheumatologische Patient:innen eigenständig zu versorgen. Digitalisierung wird bereits von Hausärzt:innen in der Versorgung von rheumatologischen Patient:innen eingesetzt, es eröffnen sich noch zahlreiche Unterstützungsmöglichkeiten für digitale Tools.

**Zusatzmaterial online:**

Die Online-Version dieses Beitrags (10.1007/s00393-025-01754-5) enthält die Abb. S1–S5, die Tab. S1–S3, den Studienfragebogen und die CHERRIES-Checkliste.

## Abstract (en)

**Introduction:**

Demographic change is expected to further aggravate the existing shortage of rheumatological care in Germany, particularly in rural regions. Increased involvement of general practitioners (GPs) and more digitalization are therefore considered promising strategies to improve care of rheumatological patients.

**Objectives:**

Assessment of the current state of GP-based care for patients with rheumatic diseases, with a particular focus on the role of digitalization.

**Materials and methods:**

To answer these questions, a cross-sectional study was conducted among GPs in the region of the Rheumatology Centre Würzburg using a questionnaire with 10 domains and 13 question items.

**Results:**

In all, 92 GPs participated in the survey, representing approximately 10% of the GPs working in Lower Franconia. On average, 3.9 patients with suspected rheumatic disease are seen per quarter and 22 patients with confirmed inflammatory rheumatic disease are medically cared for. Furthermore, 62% are confident that they can take responsibility for the care of stable rheumatological patients without rheumatological guidance. The majority of them (94.6 %) already obtain their information from digital sources. Only 30.4 % are familiar with digital training for rheumatology, and only 3% uses screening tools for rheumatism. Digital health applications (DiGAs) have already been prescribed by 61%.

**Conclusion:**

GPs are already making a significant contribution to the care of rheumatological patients and they are willing to provide care for rheumatological patients with stable pharmacotherapy. Digitalization is already being used by GPs in the care of rheumatology patients, but there is still considerable potential for use of digital tools/applications.

## Hintergrund und Fragestellung

Der bereits jetzt bestehende Mangel an rheumatologischen Versorgungskapazitäten wird in den kommenden Jahren noch weiter zunehmen. Daher werden neue Versorgungskonzepte benötigt. Das jüngste Memorandum der Deutschen Gesellschaft für Rheumatologie und Klinische Immunologie (DGRh) schlägt hier die vermehrte Einbindung von Hausärzt:innen und mehr Digitalisierung als mögliche Lösungsansätze vor. In einer Querschnittsstudie in Unterfranken wollten wir daher den Status quo der hausärztlichen Versorgung von rheumatologischen Patient:innen und die Verwendung digitaler Tools in diesem Kontext untersuchen.

Die rheumatologische Versorgung in Deutschland steht in den kommenden Jahren vor großen strukturellen Herausforderungen, die sich aus dem fortschreitenden demografischen Wandel ergeben [1]: Im jüngsten Memorandum der DGRh wird dargestellt, wie sich eine bereits jetzt bestehende Unterversorgung rheumatologischer Patient:innen weiter verschärfen wird: Ursächliche Faktoren sind zum einen demografisch bedingt steigende Fallzahlen und zum anderen ein vermehrter Renteneintritt rheumatologischer Fachärzt:innen bei abnehmendem Nachwuchs sowie ein stattfindender Abbau stationärer Versorgungskapazitäten [1]. Besonders ländliche Regionen sind hiervon betroffen [1]. Daher werden neue Versorgungskonzepte benötigt, um diese Herausforderungen zu meistern. Die DGRh schlägt den Einsatz von digitalen Konzepten in Versorgung und Weiterbildung vor [1]. Auch die European Alliance of Associations for Rheumatology (EULAR) empfiehlt den vermehrten Einsatz von Remote Care und Telehealth zur Verbesserung der Versorgungssituation [2]. Eine weitere Möglichkeit zur Bewältigung der Unterversorgung, die ebenfalls im Memorandum diskutiert wird, ist die bessere Vernetzung mit Hausärzt:innen sowie deren gezielte Schulung und Umgang mit rheumatologischen Patient:innen [1]. Dies haben wir zum Anlass genommen, um die hausärztliche Versorgungsrealität von Patient:innen mit rheumatischen Erkrankungen in einer ländlichen Region wie Unterfranken, die besonders von Unterversorgung bedroht ist, zu erfassen. Weiterhin sollte untersucht werden, an welcher Stelle digitale Konzepte bereits jetzt in hausärztlicher Weiterbildung und Versorgung verwendet werden. Hierzu sollte eine explorative Erhebung unter den Hausärzt:innen im Versorgungsgebiet des Rheumazentrums Würzburg erfolgen.

## Untersuchungsmethoden

### Studiendesign und Datenquelle

Diese Querschnittsstudie war als anonyme Umfrage unter hausärztlich tätigen Ärzt:innen im Versorgungsgebiet des Rheumazentrums Würzburg/der rheumatologischen Ambulanz des Universitätsklinikums Würzburg konzipiert. Die Datenerhebung fand vom 22.02.2025 bis 06.05.2025 statt. Hierfür wurde ein selbsterstellter Fragebogen mit insgesamt 10 Domänen und 13 Fragenitems verwendet (der Fragebogen findet sich im elektronisches Zusatzmaterial online). Der Fragebogen wurde mittels Google Forms umgesetzt. Die CHERRIES-Checkliste zur Umfrage befindet sich im Zusatzmaterial.

### Stichprobe

Über die Jahrestagung des Rheumazentrums Würzburg am 22.02.2025 sowie postalisch über eine, dem Arztbrief gemeinsam betreuter Patient:innen beigefügte Einladung zur Teilnahme an der Umfrage und eine hausärztliche WhatsApp-Gruppe wurden Ärzt:innen zur freiwilligen und anonymen Teilnahme an der Online-Umfrage rekrutiert. Die Teilnehmenden der Tagung erhielten den Fragebogen in Papierform. Berücksichtigt wurden ausschließlich Rückmeldungen aus der hausärztlichen Versorgung.

### Ethik

Da es sich um eine anonyme Umfrage ohne personenbezogene Daten handelt, war keine Stellungnahme einer Ethikkommission notwendig.

### Statistische Analyse

Zur Berechnung von Mittelwerten und Medianen wurde Excel (Microsoft Office LTSC Professional Plus 2024) verwendet. Die grafische Darstellung der Ergebnisse erfolgte mittels PowerPoint (Microsoft Office LTSC Professional Plus 2024). Häufigkeitsunterschiede wurden auf statistische Signifikanz mittels des Chi-Square Tests untersucht. Ein *p*-Wert von < 0,05 wurde hierbei als signifikant betrachtet. Zur Berechnung wurde Prism Version 5.0 verwendet.

## Ergebnisse

### Deskriptive Ergebnisse

Insgesamt nahmen 92 Ärzt:innen in der hausärztlichen Versorgung an der Umfrage teil. Hiervon waren 80,4 % (*n* = 74) aus dem Fachbereich Allgemeinmedizin und 19,6 % (*n* = 18) aus der Inneren Medizin. Das Durchschnittsalter lag bei 51,3 Jahren (Median: 48 Jahren, zur Altersverteilung siehe ergänzende Abb. S1 im Zusatzmaterial).

### Hausärztliche Versorgungsrealität von rheumatologischen Patient:innen in Unterfranken

**Abb. 1:**
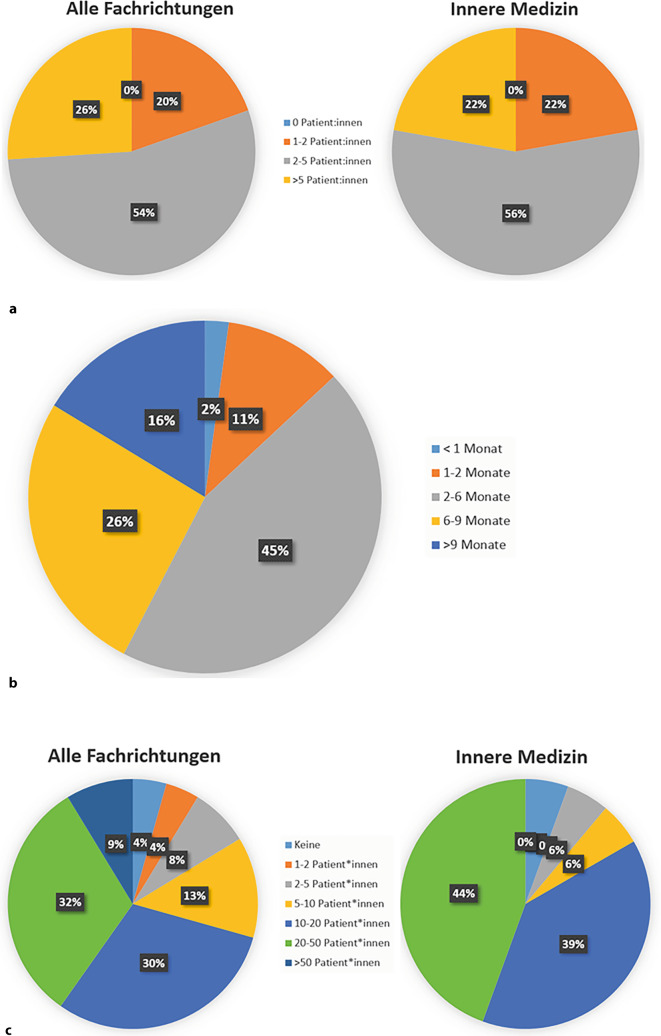
Hausärztliche Versorgung von rheumatologischen Patient:innen in Unterfranken. **a** Patient:innenaufkommen mit V. a. eine entzündlich-rheumatische Erkrankung im Quartal. **b** Wartezeit bis zur rheumatologischen Vorstellung. **c** Mitbetreute Patient:innen mit entzündlich-rheumatischer Erkrankung

**Abb. 2:**
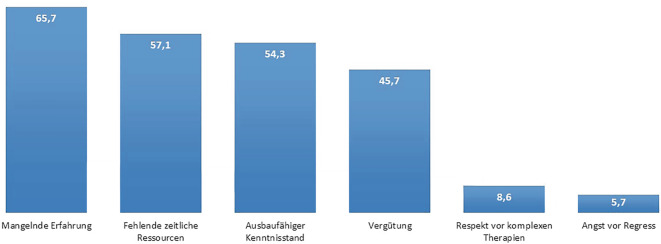
Gründe für mangelnde Möglichkeit zur eigenverantwortlichen Betreuung von Patient:innen mit entzündlich-rheumatischer Erkrankung in Prozent

Die Teilnehmenden sehen im Mittel 3,9 (Median: 3,5) Patient:innen mit V. a. eine entzündlich-rheumatische Erkrankung pro Quartal (zur Verteilung siehe Abb. 1a). Hierbei gibt es keine Unterschiede zwischen Allgemeinmediziner:innen und Internist:innen. Die Dauer bis zur rheumatologischen Erstvorstellung liegt im Durchschnitt bei 5,5 Monaten (Median: 4 Monate; Abb. 1b). Im Durchschnitt werden 22,1 Patient:innen mit rheumatischer Erkrankung durch die Hausärzt:innen mitbehandelt (Median: 15). Unterschiede zwischen Internist:innen und Allgemeinmediziner:innen zeigen sich erneut nicht (beide Gruppen im Durchschnitt 22 Patient:innen/Median: 15; Abb. 1c). 62 % der Befragten geben an, dass sie sich auch eine eigenverantwortliche Betreuung von Patient:innen mit stabil eingestellter rheumatoider Arthritis (RA) oder Spondyloarthritiden (SpA) ohne mitbetreuende Rheumatolog:in zutrauen würden (Abb. S2). Erneut zeigen sich keine Unterschiede zwischen den Fachrichtungen, und auch das Alter hatte keinen Einfluss. Auf die Frage, warum sich die übrigen 38 % keine Behandlung zutrauen würden, werden an erster Stelle mangelnde Erfahrung, dann zeitliche Ressourcen und an dritter Stellte ein ausbaufähiger Kenntnisstand genannt. Erst danach folgen finanzielle Aspekte wie Vergütung oder Angst vor Regressen (Abb. 2). Nummerisch zeigten sich zwar altersabhängig unterschiedliche Gründe für die fehlende Bereitschaft zur eigenverantwortlichen Betreuung, jedoch waren diese nicht statistisch signifikant (Tab. S1 im Zusatzmaterial).

### Aktueller Einfluss der Digitalisierung auf die hausärztliche Versorgung

**Abb. 3:**
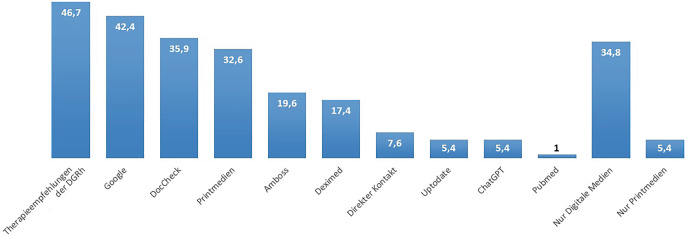
Informationsquellen zur Diagnostik und Therapie von RA- und SpA-Patient:innen in Prozent

**Abb. 4:**
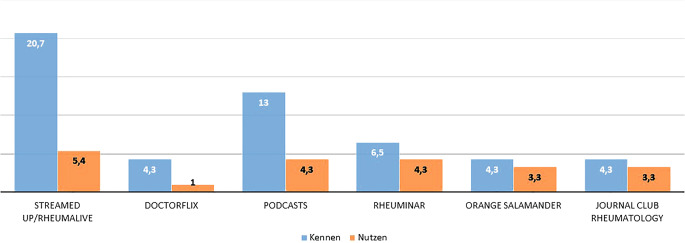
Bekanntheit und Nutzung von digitalen Fortbildungsangeboten zur Rheumatologie in Prozent

**Abb. 5:**
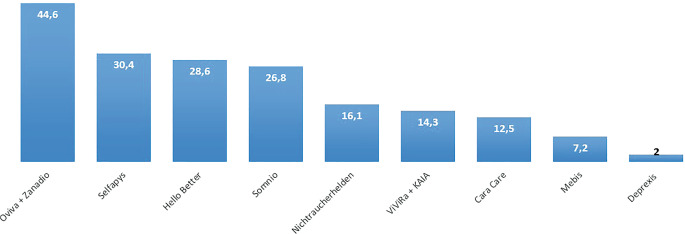
Häufigkeit der verordneten DiGAs in Prozent. Bei HelloBetter wurde nur nach HelloBetter Stress und Burnout, Schlafen und chronische Schmerzen gefragt

Von den befragten Hausärzt:innen geben 34,8 % (*n* = 32) an, dass sie ausschließlich digitale Informationsquellen zur Behandlung ihrer RA- und SpA-Patient:innen nutzen. Dem gegenüber stehen lediglich 5,4 % (*n* = 5), welche ausschließlich Printmedien nutzen. Der größte Teil (*n* = 43, 46,7 %) nutzt die Therapieempfehlungen der DGRh. Large Language Models (LLM) und künstliche Intelligenz (KI) wie ChatGPT werden nur von 5,4 % (*n* = 5) eingesetzt. Abb. 3 listet die hauptsächlich genutzten Informationsquellen auf. Altersabhängige Unterschiede in der Präferenz von Printmedien waren nicht statistisch signifikant (Tab. S2). Die Online-Verfügbarkeit von Therapieempfehlungen und Leitlinien wird mit dem Notendurchschnitt 3,15 (Median 3) bewertet (Abb. S3). Nur 3 % der Hausärzt:innen in Unterfranken nutzen ein digitales Tool zum Screening auf eine rheumatische Erkrankung (*n* = 3) (Abb. S4). Ein ähnliches Bild zeigt sich auch bei digitalen Fortbildungsangeboten zur Rheumatologie: 69,6 % (*n* = 64) kennen keine der genannten digitalen Fortbildungsangebote (Abb. 4). Am bekanntesten war Streamed-up/Rheumalive, welches knapp ein Fünftel der Befragten kennt. Die weiteren Informationen stellt Abb. 4 dar. Die Kenntnis von digitalen Fortbildungsangeboten unterschied sich nicht zwischen den Altersgruppen (Tab. S3). Digitale Gesundheitsanwendungen (DiGAs) werden bereits von 60,9 % (*n* = 56) rezeptiert (Abb. S5). Zu den häufigsten rezeptierten DiGAs zählen dabei DiGAs für Adipositas, Depression und Angststörung, chronische Schmerzen und Schlafstörungen. Abb. 5 gibt einen Überblick über die Häufigkeit der verordneten DiGAs.

## Diskussion

In dieser Querschnittsstudie wurde die aktuelle hausärztliche Versorgungsrealität von rheumatologischen Patient:innen im Bereich Unterfranken erfasst und untersucht, welchen Beitrag hierbei die Digitalisierung bereits leistet. An unserer Umfrage nahmen 92 hausärztlich tätige Ärzt:innen teil. Dies entspricht bei einer Personenanzahl von 963 tätigen Hausärzt:innen in Unterfranken einem Anteil von knapp 10 % [3]. Das Durchschnittsalter der praktizierenden Hausärzt:innen in Unterfranken liegt nach Angaben der Kassenärztlichen Vereinigung bei 54,9 Jahren, in unserer Stichprobe bei 51,3 Jahren [3]. Damit kann aus unserer Sicht unsere per Zufall erhobene Stichprobe als aussagekräftig für die unterfränkischen Hausärzt:innen betrachtet werden.

Daten zur Inzidenz und Prävalenz entzündlich-rheumatischer Erkrankungen in Unterfranken liegen nicht vor, können jedoch aus deutschlandweiten bzw. mitteleuropäischen Zahlen abgeleitet werden: Die Inzidenz der RA beträgt etwa 20–50 Fälle pro 100.000 Einwohner jährlich [4]. SpA und RA weisen in Deutschland vergleichbare Prävalenzen auf (RA: 0,8–1,2 %, SpA: 1,0–1,4 %), mit einer Gesamtprävalenz aller entzündlich-rheumatischen Erkrankungen von etwa 3 % [5]. Bei 1,3 Mio. Einwohnern in Unterfranken entspricht das ca. 1300 Neuerkrankungen pro Jahr an RA/SpA bzw. 2000 bei Einbezug aller Formen [3]. Umgerechnet auf etwa 1000 Hausärzt:innen ergibt das rund 1,3 bzw. 2 Neuerkrankungen pro Ärzt*in und Jahr [3]. Die in unserer Studie berichteten durchschnittlich 3,9 Verdachtsfälle pro Quartal erscheinen daher plausibel. Mit durchschnittlich 5,5 Monaten Wartezeit bis zur Erstvorstellung bei der Rheumatolog:in in unserer Umfrage scheinen sich die noch 2018 berichteten 3 Monate im ländlichen Raum noch einmal deutlich verlängert zu haben [1]. In einer zwischen September 2019 und April 2021 durchgeführten Umfrage unter Patient:innen in Franken lag die mediane Wartezeit bis zur rheumatologischen Erstvorstellung sogar bei 30 Wochen/7,5 Monaten [6]. Bei einer geschätzten Gesamtprävalenz von 2 % für RA und SpA ist in Unterfranken mit etwa 26.000 RA- und SpA-Patient:innen zu rechnen. Verteilt auf rund 1000 hausärztlich tätige Ärzt*innen ergibt sich daraus ein Mittelwert von etwa 26 Patient:innen pro Ärzt*in – ein Wert, der nahezu mit den im Rahmen unserer Umfrage angegebenen 22 Patient:innen pro Praxis übereinstimmt [3, 5]. Bei geschätzt mindestens einer Vorstellung pro Quartal pro Patient:in sieht wahrscheinlich jede Hausärzt:in in Unterfranken mehrfach pro Woche eine Patient:in mit einer rheumatischen Erkrankung in der Vorgeschichte in der eigenen Praxis. Damit tragen die Hausärzt:innen bereits jetzt erheblich zur Mitversorgung rheumatologischer Patient:innen bei. Dies entspricht auch der Selbstwahrnehmung von Hausärzt:innen zu ihrer Rolle in der Versorgung von rheumatologischen Patient:innen in der französischen GEPRA-I-Studie [7]. Für Deutschland gibt es hierzu keine vergleichbaren Daten. Umso interessanter ist es, dass sich 60 % der befragten Hausärzt:innen auch zutrauen würden, Patient:innen mit unkomplizierter und stabil eingestellter RA oder SpA eigenverantwortlich zu versorgen. Zu einer solchen Einschätzung liegen uns aktuell keine vergleichbaren Daten aus dem deutschsprachigen Raum vor. In einer vergleichbaren amerikanischen Umfrage unter Hausärzt:innen waren immerhin 80 % bereit dazu, Basistherapeutika selbstständig zu verordnen, und 40 % sogar dazu, auch Basistherapeutika neu zu beginnen [8]. Zum eigenständigen Treffen von diagnostischen Entscheidungen war die Bereitschaft ebenfalls sehr hoch [8]. In der oben bereits erwähnten GEPRA-I-Studie sahen die befragten französischen Hausärzt:innen ihre Aufgabe mehr in der Diagnostik und Überwachung als in der Therapieeinleitung, zumal hier relevante Wissenslücken angegeben wurden [7]. Daher scheint es umso erstaunlicher, dass in unserer Umfrage ein Großteil der Hausärzt:innen zur selbstständigen Betreuung bereit war. Möglicherweise gingen die Befragten an dieser Stelle davon aus, dass es sich um Patient:innen mit stabiler Basistherapie handelt, die bereits durch eine Rheumatolog:in gesehen und eingestellt wurden und um keine Erstdiagnosen. An dieser Stelle kann dank der Fortschritte im Bereich rheumatologischer Telehealth bzw. Remote Care, auch in Zusammenarbeit mit Hausärzt:innen, sogar diskutiert werden, ob und in welchem Rahmen stabil eingestellte Patient:innen in Zukunft überhaupt noch von Rheumatolog:innen vor Ort gesehen werden müssen [9, 10]. Inwiefern Studien in anderen Gesundheitswesen mit unterschiedlichen ärztlichen Ausbildungen auf das deutsche System übertragbar sind, bleibt an dieser Stelle aber unklar. Bei den 38 %, die sich keine eigenverantwortliche Betreuung ohne Rheumatolog:in zutrauen, zeigten sich aber die gleichen Gründe wie in den internationalen Erhebungen: an erster Stelle mangelnde Erfahrung und ein ausbaufähiger Kenntnisstand [7, 8].

Eine effektive Weiterbildung von Hausärzt:innen hinsichtlich rheumatischer Erkrankungen scheint eine Möglichkeit zu sein, die hausärztliche Versorgung rheumatologischer Patient:innen zu unterstützen. Daher wurde im Rahmen der Befragung ermittelt, welche Informationsquellen Hausärzt:innen für das Management und die Therapie rheumatischer Erkrankungen, insbesondere SpA und RA, nutzen. Auf Platz 1 wurden hier die offiziellen Therapieempfehlungen der DGRh genannt. Auf den weiteren Plätzen folgten vor allem digitale Quellen. Aber immerhin ein Drittel der Befragten griff noch auf Printmedien wie Bücher zurück, eine ausschließliche Nutzung von Printmedien spielt jedoch keine Rolle mehr. Während die amerikanischen Hausärzt:innen ihre Informationen in einer vergleichbaren Umfrage von 2011 noch zum Großteil aus Printmedien und nur nachranging aus Onlinequellen bezog, scheint sich nun eine eindeutige Trendwende hin zu digitalen Quellen vollzogen zu haben [8]. Die Verwendung von offiziellen Materialen der DGRh scheint hierbei besonders hilfreich zu sein, da diese regelmäßig aktualisiert und an den aktuellen Kenntnisstand angepasst werden. In der 2019 durchgeführten GEPRA-I-Studie gaben knapp 92 % der befragten französischen Hausärzt:innen an, dass sie nicht über aktualisierte Therapieempfehlungen informiert würden [7]. Trotz dieser Präferenz digitaler Angebote wurde die Online-Verfügbarkeit von Therapieempfehlungen nur mit dem Notendurchschnitt 3,15 bewertet. Hier gibt es also sicher noch Verbesserungspotenzial.

LLMs und KI werden von den Hausärzt:innen als Informationsquelle noch kaum eingesetzt, obwohl bereits mittels Fallvignetten gezeigt wurde, dass ChatGPT durchaus zur korrekten Entscheidungsfindung bei Therapie und Management von rheumatischen Erkrankungen beitragen kann, wenn auch noch nicht auf dem gleichen Niveau wie rheumatologische Expertenteams [11].

Hier würde also Potenzial bestehen, die hausärztliche Versorgung mittels KI zu unterstützen. KI kann auch beim Screening und bei der Abklärung auf eine rheumatische Erkrankung helfen. Hierfür stehen bereits vielversprechende Tools wie Symptomchecker zur Verfügung, deren Treffsicherheit aber noch verbesserungswürdig erscheint [12, 13]. Neuere Arbeiten unter Verwendung der gängigen LLMs zeigen jedoch, dass moderne LLMs „klassische“ Symptomchecker in der diagnostischen Genauigkeit bereits überholt haben und auch Rheumatolog:innen nicht mehr unterlegen zu sein scheinen [14, 15]. Symptomchecker und LLMs werden von Hausärzt:innen in Unterfranken de facto für diesen Zweck aber noch nicht genutzt. Bei rechnerisch auf Basis dieser Umfrage etwa 4000 Verdachtsfällen auf eine rheumatische Erkrankung pro Quartal bestünde hier weiteres Potenzial, die Versorgung zu optimieren. Von den unterfränkischen Hausärzt:innen, die sich eine eigenverantwortliche Betreuung von Patient:innen mit entzündlich-rheumatischer Erkrankung nicht zutrauen, wurden als Hauptgründe mangelnde Erfahrung und ein ausbaufähiger Kenntnisstand genannt. Dies deckt sich auch mit den vergleichbaren internationalen Erhebungen unter Hausärzt:innen [7, 8]. Trotz des selbst eingeschätzten ausbaufähigen Kenntnisstandes werden aktuell aber kaum digitale Fortbildungsangebote genutzt, da die meisten abgefragten Formate nur einem Bruchteil der unterfränkischen Hausärzt:innen bekannt sind. Demgegenüber steht die Tatsache, dass rund 20 der 92 Hausärzt:innen über unsere Tagung in Präsenz rekrutiert wurden. Damit ergibt sich auch hier erhebliches Potenzial, die Hausärzt:innen für die Versorgung von Rheuma-Patient:innen besser zu sensibilisieren und zu schulen, besonders in ländlichen Regionen wie Unterfranken mit weiten Distanzen zu entsprechenden Zentren mit Fortbildungsangeboten. DiGAs werden dagegen bereits breit von 60 % der befragten Hausärzt:innen verordnet. In einer Umfrage der Stiftung Gesundheit von 2023 gaben bereits damals fast 50 % der befragten Hausärzt:innen an, dass sie DiGAs rezeptieren würden [16]. Ein Trend der sich im letzten Jahr sicher noch einmal verstärkt haben dürfte, da die DiGA-Verordnungszahlen weiter zugenommen haben [17]. Dagegen gaben in einer Umfrage unter Internist:innen nur 31 % an, eine DiGA schon einmal verschrieben zu haben [18]. Die Befragten waren aber größtenteils in Kliniken und damit in einem anderen Setting tätig, was den Unterschied zu den Ergebnissen der hausärztlich tätigen Ärzt:innen in unserer Umfrage erklären könnte [18]. Damit sind die berichteten 60 % in unserer Umfrage sicherlich als realistisch für das generelle DiGA-Verordnungsverhalten von Hausärzt:innen zu sehen und zeigen deren zunehmend positive Einstellung gegenüber dieser neuen Therapieform. Laut DiGA-Report 2024 sind die häufigsten von Hausärzt:innen verordneten DiGAs aus den Indikationsgebieten Stoffwechsel und Psychologie, was sich mit unseren Ergebnissen deckt [17]. Da aktuell jedoch noch keine DiGA für eine rheumatologische Indikation vorliegt, ist deren Einsatz aktuell vor allem für Komorbiditäten denkbar [19, 20]. Jedoch könnten auch bald erste DiGAs für rheumatologische Indikationen zugelassen werden und damit die Versorgung von rheumatologischen Patient:innen durch Hausärzt:innen aktiv unterstützen [21, 22].

### Limitationen

Die Limitation unserer Umfrage besteht darin, dass keine strukturierten Interviews durchgeführt wurden, sondern die Teilnehmenden sich selbstständig mit den Fragen auseinandersetzen mussten, was zu Missverständnissen bei bestimmten Fragen geführt haben könnte. Weiterhin wurde ein selbsterstellter Fragebogen verwendet, welcher bisher nicht validiert wurde. Allerdings steht derzeit nach unserem Kenntnisstand kein Fragebogen zur Verfügung, der die Inhalte unserer Befragung im gleichen Maße abdeckt. Weiterhin erfolgte die Teilnahme freiwillig und unselektiert per Zufall, sodass nicht ausgeschlossen werden kann, dass auch mehrere Hausärzt:innen aus der gleichen Praxis teilgenommen haben, was natürlich die Repräsentativität der Daten einschränken würde. Ein Selektionsbias von besonders an rheumatischen Erkrankungen interessierten Hausärzt:innen ist ebenfalls nicht auszuschließen. Weiterhin wurde der Umfang des Fragebogens bewusst im Sinne einer hohen Teilnahmebereitschaft der Hausärzt:innen prägnant und konzis gehalten, sodass bei den Auswahlfragen (z. B. zur DiGA-Nutzung) Vorauswahlen durch die Autor:innen getroffen werden mussten, was gewisse Verzerrungen bedingen kann. Nichtsdestotrotz sind wir der Meinung, dass die Aussagekraft und die Einmaligkeit der Inhalte dieser Befragung für die Stärke dieser Studie sprechen und ein gutes Bild über den aktuellen Versorgungsstand und dessen Potenzial in ländlichen Regionen darstellt. Insbesondere die hohe Bereitschaft, rheumatologische Patient:innen selbstständig zu versorgen, scheint ein großes Potenzial für die Zukunft darzustellen.

## Fazit für die Praxis

Eine alleinige Versorgung von rheumatologischen Patient:innen durch die Hausärzt:innen gänzlich ohne Kontakt zu Rheumatolog:innen soll nicht angestrebt werden, zumal gebietsweise eine drastische Unterversorgung mit Hausärzt:innen besteht. Möglicherweise kann jedoch die Ressource Rheumatolog:in durch verlängerte Kontrollintervalle und mehr eigenständige Entscheidung der Hausärzt:innen für ausgewählte Patient:innen geschont werden. Digitalisierung und KI scheinen hierbei vor allem in der Versorgung und Weiterbildung großes Potenzial zu haben, das aktuell noch nicht ausreichend genutzt zu werden scheint. Bis auf DiGAs sind entsprechende Tools noch wenig bekannt und kaum genutzt, was durch gezielte Kampagnen mit einfachen Mitteln leicht gefördert werden kann. Damit können auch in ländlichen Räumen wie Unterfranken die Ansätze des Memorandums der DGRh zügig in die Tat umgesetzt werden, um die Versorgung rheumatologischer Patient:innen zeitnah zu verbessern.

## Supplementary Information

ESM: Abb. S1–S5; Tab. S1–S3; Fragebogen; CHERRIES-Checklist

## Data Availability

Die Rohdaten dieser Studie sind nicht öffentlich zugänglich. Begründete Anfragen bzgl. der Rohdaten können an die Autoren gerichtet werden.
