# Supplementary material for: Survey on primary care for rheumatology patients in Lower Franconia and the contribution of digital health
Source: Z Rheumatol. 2025 Dec 1;85(3):179–87. [Article in German] doi: 10.1007/s00393-025-01754-5 (PMC13021800; doi:10.1007/s00393-025-01754-5)
Supplement: Supplementary file 1 — ESM: Abb. S1–S5; Tab. S1–S3; Fragebogen; CHERRIES-Checklist [file 393_2025_1754_MOESM1_ESM.docx]

**Supplementary File zu**

**Umfrage zur hausärztlichen Versorgungsrealität bei rheumatologischen Patient:innen in Unterfranken und zum Beitrag der Digitalisierung**

["Survey on primary care for rheumatology patients in Lower Franconia and the contribution of digital health”]

Autoren: Patrick-Pascal Strunz ^1^, Michael Gernert ^1^, Lea-Kristin Nagler ^1^, Hannah Labinsky ^1^, Matthias Fröhlich ^1^, Maurice Stetter ^2^, Clara Stetter ^1^, Lotte Possler ^3^, Marc Schmalzing^1^

Autorzugehörigkeit:

^1^ Universitätsklinikum Würzburg, Medizinische Klinik II, Rheumatologie/ Immunologie

^2^ Sanitätsdienst der Bundeswehr, Sanitätszentrum Veitshöchheim

^3^ Main-Klinik Ochsenfurt, Abteilung für Innere Medizin


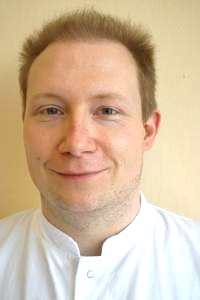
**Korrespondenzanschrift:**

PD. Dr. med. Patrick-Pascal Strunz

Medizinische Klinik II

Rheumatologie/ Klinische Immunologie

Oberdürrbacher Straße 6

97080 Würzburg

Email: [Strunz_p@ukw.de](mailto:Strunz_p@ukw.de)

Telefon:0931-201 40100

**1. Ergänzende Abbildungen:**


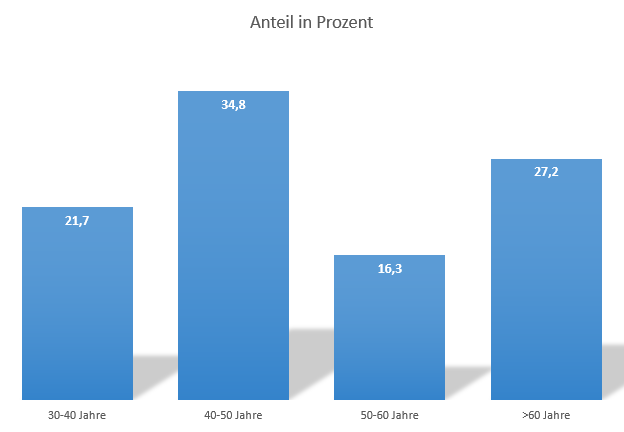


**Abbildung S1: Altersverteilung der teilnehmenden Ärzt:innen**


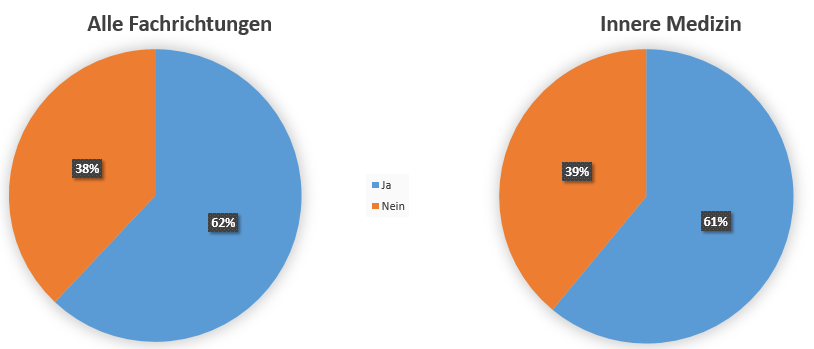

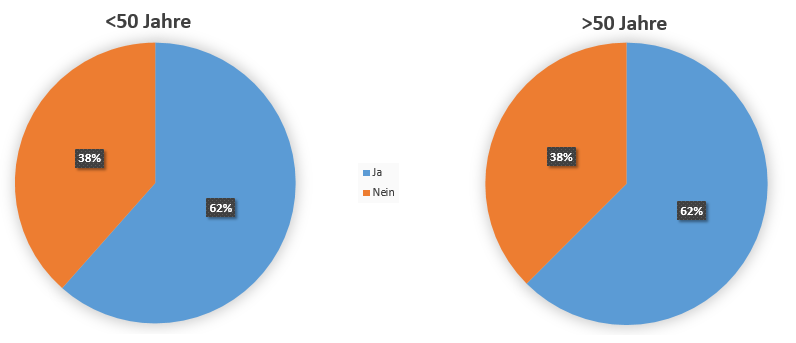


**Abbildung S2: Bereitschaft zur eigenverantwortlichen Behandlung von unkomplizierten rheumatologischen Patient:innen durch Hausärzt:innen**


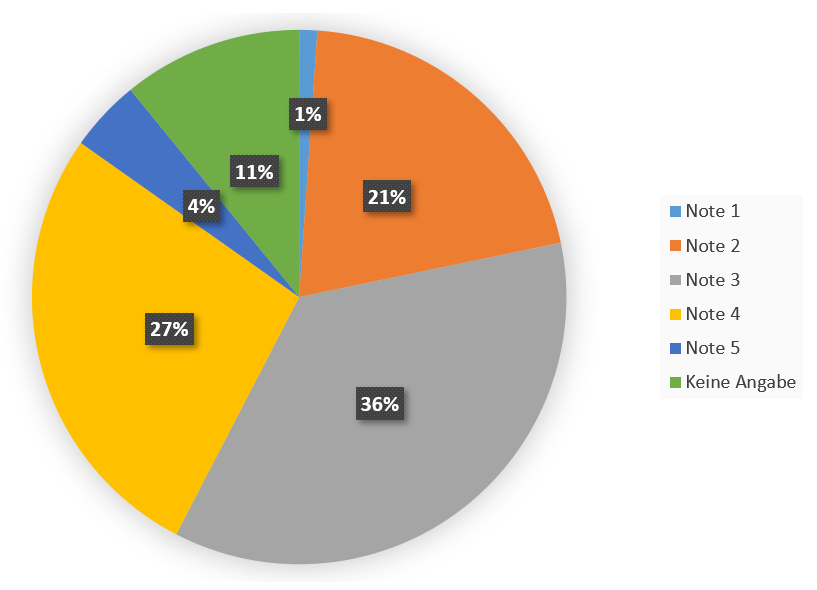


**Abbildung S3: Schulnotenverteilung für die digitale Verfügbarkeit von Leitlinien**


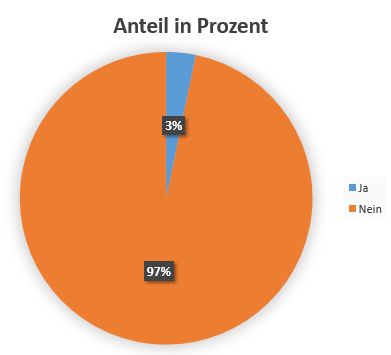


**Abbildung S4: Einsatz von digitalen Screening-Tools für rheumatische Erkrankungen**


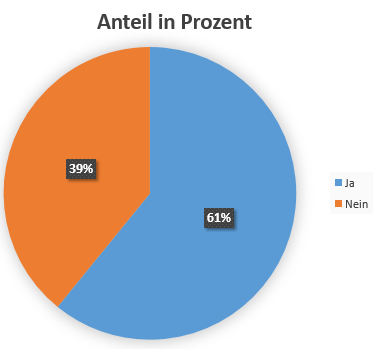


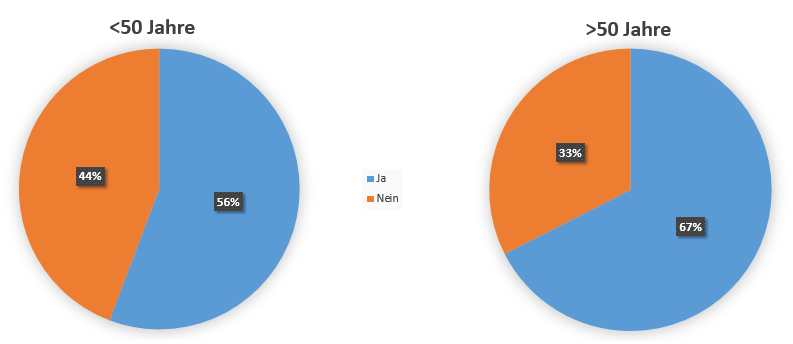


**Abbildung S5: Verordnung einer DiGA in der hausärztlichen Praxis**

**2. Ergänzende Tabellen**

|  | **Alter <50 Jahren (n=20)** | **Alter >50 Jahren (n=15)** | **p-Wert im Chi-Square Test** | **Gesamt (n=35)** |
| --- | --- | --- | --- | --- |
| Vergütung | 60% (n=12) | 26,7% (n=4) | 0,05 | 45,7% (n=16) |
| Fehlende zeitliche Ressourcen | 45% (n=9) | 73,3% (n=11) | 0,09 | 57,1% (n=20) |
| Ausbaufähiger Kenntnisstand | 55% (n=11) | 53,3% (n=8) | 0,92 | 54,3% (n=19) |
| Mangelnde Erfahrung | 60% (n=12) | 73,3% (n=11) | 0,41 | 65,7% (n=23) |
| Angst vor den Therapien | 15% (n=3) | 0% (n=0) | 0,12 | 8,6% (n=3) |
| Angst vor Regress | 10% (n=2) | 0% (n=0) | 0,21 | 5,7% (n=2) |

**Tabelle S1: Gründe für fehlende Bereitschaft zur eigenverantwortlichen Versorgung von rheumatologischen Patient:innen in Abhängigkeit vom Alter**

|  | **Alter <50 Jahren (n=52)** | **Alter >50 Jahren (n=40)** | **p-Wert im Chi-Square Test** |
| --- | --- | --- | --- |
| Ausschließlich Printmedien (n=5) | 1,9% (n=1) | 10% (n=4) | 0,09 |
| Ausschließlich digitale Quellen (n=32) | 38,5% (n=20) | 30% (n=12) | 0,40 |

**Tabelle S2: Ausschließliche Verwendung von digitalen Medien und Printmedien in Abhängigkeit des Alters**

| **Altersgruppe:** | **n/N** | **Prozentsatz** |
| --- | --- | --- |
| 30-40 Jahre | 7/20 | 35% |
| 40-50 Jahre | 9/32 | 28,1% |
| 50-60 Jahre | 4/15 | 26,7% |
| >60 Jahre | 8/25 | 32% |
| Gesamt | 28/92 | 30,4% |

**Tabelle S3 : Kenntnis von digitalen Fortbildungsangeboten in Abhängigkeit des Alters**

**3. Fragebogen:**

**Gegenwärtige Patientenströme und Versorgungsstruktur von Patienten mit rheumatischen Erkrankungen sowie der Einsatz digitaler Tools in der Patientenversorgung im Bereich des Rheumazentrums Würzburg.**

**1. Alter**

Bitte geben Sie Ihr Alter an:

30-40 Jahre

40-50 Jahre

50-60 Jahre

>60 Jahre

**2. Fachbereich**

Welcher Fachrichtung gehören Sie an?

Allgemeinmedizin

Orthopädie

Innere Medizin in hausärztlicher Versorgung

Innere Medizin (außer Rheumatologie)

Andere Fachdisziplin: _____________________________

**3. Patienten**

Wie viele Patienten mit Verdacht auf eine rheumatische Erkrankung sehen Sie im Quartal?

Keine >5

1-2 2-5

**4. Termin**

Wie lange dauert es im Schnitt, bis diese Patienten einen Termin bei einer Rheumatologin/ einem Rheumatologen bekommen?

< 1 Monat 2-6 Monate

1-2 Monate 6-9 Monate

> 9 Monate

**5. Mitbetreute Rheuma-Patientinnen und Patienten**

Wie viele Patientinnen und Patienten mit einer rheumatoiden Arthritis (RA) oder axialen Spondyloarthritis/ Morbus Bechterew betreuen Sie **in Kooperation** mit einer Rheumatologin/ einem Rheumatologen in Ihrer Praxis/ Einrichtung?

Keine 10-20

1-2 20-50

2-5 >50

5-10

**6. Versorgung von Rheuma-Patientinnen und Patienten (internistisch)?**

6.1 Könnten Sie sich vorstellen, Patientinnen und Patienten mit einer stabil eingestellten rheumatoiden Arthritis oder axialen Spondyloarthritis/ Morbus Bechterew größtenteils **ohne Mitbetreuung** durch eine internistische Rheumatologin oder Rheumatologen dauerhaft zu versorgen, wenn entsprechende Informationen zur Therapie und Therapie-Monitoring vorhanden wären?

Ja

Nein

6.2 Falls Sie mit **Nein** geantwortet haben, was sind die Gründe für Ihre Entscheidung?

Keine Zeit

Vergütung

Mangelnde Erfahrung

Ausbaufähiger Kenntnisstand

Andere: __________________

**7. Digitaler Zugang zu deutschsprachigen Fachinformationen/ Leitlinien/etc.**

7.1 Wenn Sie Informationen zur Diagnostik oder Behandlung einer rheumatoiden Arthritis oder axialen Spondyloarthritis/ Morbus Bechterew benötigen, auf welche Informationsquellen greifen Sie zurück?

Google

ChatGPT

DocCheck

Therapieempfehlungen der DGRh

Uptodate

Printmedien

Andere:________________________

7.2 Wie würden Sie die Verfügbarkeit von offiziellen Therapieempfehlungen via Internet oder Apps beurteilen?

Sehr schlecht/ stark verbesserungswürdig (Schulnote 5)

Schlecht/ verbesserungswürdig (Schulnote 4)

Befriedigend/ Geringes Verbesserungspotenzial (Schulnote 3)

Gut/ kein Verbesserungsbedarf (Schulnote 2)

Sehr gut/ übertrifft meine Erwartungen und Bedarf (Schulnote 1)

Ich nutze Leitlinien und Fachinformationen nur in Printform/ kann die Frage nicht beantworten

**8. Digitale Tools zum Screening auf Rheuma**

Es gibt verschiedene digitale Screening-Tools wie Rheuma-Check, Bechterew-Check der Charité oder Rheport von der RHADAR Gruppe. Haben Sie ein solches Tool schon genutzt?

Ja

Nein

**9. Digitale Fortbildungsangebote**

Welche digitalen Fortbildungsangebote zur Rheumatologie kennen und/oder nutzen Sie?

Kennen Nutzen

Streamed Up/ RheumaLive

Doctorflix

Podcasts (Z.B. Expert Talk, RheumaRadio, Rheuma nachgefragt)

Rheuminar der Rheumaakademie

App “Orange Salamander”

Journal Club Rheumatology/ jc_rhmtlgy auf youtube

Andere:_______________________

**10. DIGAs**

10.1 Haben Sie schon einmal eine Digitale Gesundheitsanwendung/ DiGA rezeptiert?

Ja

Nein

10.2 Falls Sie mit Ja geantwortet haben, welche der folgenden rezeptieren Sie am häufigsten?

KAIA (Rückenschmerzen)

HelloBetter (Schmerz, Depression, Schlaf, Stress etc.)

Cara Care (Reizdarm)

Mebis (Diabetes)

NichtraucherHelden (Sucht)

Selfapys (Schmerz, Depression, Adipositas)

Somnio (Insomnie)

Andere:___________________

**CHERRIES-Checkliste^1^**

| **CHERRIES Item** | **Angaben für diese Studie** |
| --- | --- |
| **Design** | |
| Design | Anonyme, Online‑Umfrage unter hausärztlich tätigen Ärzt:innen im Versorgungsgebiet des Rheumazentrums Würzburg. Zielpopulation: Hausärzt:innen in Unterfranken; Stichprobe: Convenience Sample. Erhebungszeitraum: 22.02.2025–06.05.2025. Plattform: Google Forms. |
| Ziele / Fragestellungen | Erfassung des Status quo der hausärztlichen Versorgung rheumatologischer Patient:innen und der Rolle der Digitalisierung. |
| **Ethik, Einwilligung und Aufklärung („IRB (Institutional Review Board) approval and informed consent process“)** | |
| Ethikvotum (IRB/REC) | Nicht erforderlich, da ausschließlich anonyme Daten erhoben wurden (deutsches Recht). |
| Aufklärung und Einverständniserklärung | Vor dem Ausfüllen des Fragebogens über Google Forms erfolgte auf der ersten Seite der Hinweis, dass es sich um eine anonyme Umfrage handelt und dass keine personenbezogenen Daten gespeichert werden. Da es sich damit um eine anonyme Datenerhebung handelt, war keine unterschriebene Einverständniserklärung nach deutschem Recht notwendig. |
| Datenschutz | Es wurden keine persönlichen oder personenbezogenen Daten erhoben. Die anonymen Ergebnisse der Studie standen nur dem Studienleiter P. Strunz passwortgeschützt zur Verfügung. Die Rohdaten wurden nicht an Dritte weitergegeben. |
| **Entwicklung und Vortestung („Development and pre-testing“)** | |
| Fragebogenentwicklung / Pretest | Selbst erstellter Fragebogen (10 Domänen, 13 Items); formale Validierung oder Pretest aufgrund des explorativen Characters nicht erfolgt und damit nicht berichtet(als Limitation genannt). |
| **Rekrutierungsprozess, Beschreibung des Kontaktweges („Recruitment process and description of the sample having access to the questionnaire”)** | |
| Rekrutierungskanäle | Einladung auf der Jahrestagung des Rheumazentrums (22.02.2025), postalische Beilage zu Arztbriefen gemeinsam betreuter Patient:innen, hausärztliche WhatsApp‑Gruppe. |
| Offene vs. geschlossene Umfrage | Gezielte Rekrutierung in einer definierten Zielgruppe (de facto geschlossene Umfrage); methodisch Convenience‑Stichprobe. |
| Kontaktmodus | Zugriff über Online‑Link (Google Forms); postalische Anschreiben enthielten den Link bzw. im Rahmen der Jahrestagung wurde der Fragebogen in gedruckter Form verteilt. |
| Bewerbung der Studie | Es fand keine Bewerbung der Studie statt, da es sich um eine geschlossene Umfrage handelte. |
| **Umfrageverwaltung („Survey administration“)** | |
| Web/Email | Die Studie war auf Google Forms gehostet. |
| Kontext / Setting | Region Unterfranken; Zielgruppe: in der hausärztlichen Versorgung tätige Ärzt:innen (Internist:innen und Allgemeinmediziner:innen). |
| Freiwilligkeit | Die Teilnahme an der Umfrage war freiwillig |
| Anreize | Es erfolgten keine gezielten Anreize zur Teilnahme. Die Teilnahme war freiwillig. Eine Vergütung erfolgte nicht. |
| Erhebungszeitraum | 22.02.2025–06.05.2025. |
| Randomisierung der Items | Es erfolgte keine Randomisierung der Items. Die Abfolge der Items war fix. Es gab keinen Unterschied zwischen gedruckter Form und digitalisierter Form des Fragebogens. |
| Adaptives Fragen | Teilweise beinhaltet der Fragebogen adapitve Fragen |
| Anzahl der Items | 13 Items in 10 Domänen. |
| Anzahl der Seiten/ Screens | 1 Screen bzw. 4 DIN A4 Seiten beidseitig bedruckt |
| Vollständigkeitsprüfung | Bei der digitalisierten Form des Fragebogens auf Google Forms waren alle Fragen bis auf die adaptiven Fragen Pflichtfragen (ohne Beantwortung dieser Fragen konnte der Fragebogen nicht abgeschickt werden). Bei der Papierform war dies nicht möglich, daher wurden nur komplette Fragebögen berücksichtigt. |
| Review und Revidierung | Die Teilnehmenden konnten die Antworten sowohl in der digitalen als auch in der Printform revidieren. |
| **Antwort Raten („Response rates“)** | |
| Eindeutigkeit der Teilnehmenden („Unique site visitor“) | Hierzu wurden keine Maßnahmen aufgrund der Anonymität der Umfrage und deren explorativen Charakters ergriffen. |
| View Rate | Dies kann aufgrund der Verwendung von Google Forms nicht angegeben werden. |
| Teilnahme Rate („Participation rate“) | Dies kann aufgrund der Verwendung von Google Forms nicht angegeben werden. |
| Vollständigkeit der Rückläufer („completion rate“) | Die completion rate ist unbekannt. Es wurden nur komplette Fragebögen abgegeben. |
| **Verhinderung mehrfacher Einträge durch dieselbe Person („Preventing multiple entries from the same individual“)** | |
| Verwendung von Cookies | Es wurden keine Cookies verwendet |
| IP check | Die IPs wurden nicht getracked |
| Log file analysis | Ein Log file wurde nicht verwendet aufgrund des anonymen Charakters der Umfrage |
| Registrierung | Da es sich um eine anonyme Umfrage handelte, mussten sich die Teilnehmenden nicht registrieren. |
| **Analyse** | |
| Umgang mit inkompletten Fragebögen | Aufgrund der Verwendung von Pflichtfragen lagen keine unvollständigen digitalen Rückläufer vor. Bei der papierbasierten Umfrage wurden nur komplette Fragebögen gewertet. |
| Atypischer Zeitstempel | Diese Analyse erfolgte nicht |
| Statistik / Datenanalyse | Deskriptive Statistik (Mittelwert/Median) und ChiQuadratTests (Signifikanzniveau p<0,05). Software: Excel (deskriptiv), Prism 5.0 (Tests); Grafiken in PowerPoint. Keine Gewichtung/Adjustierung. |

^1^ basierend auf Eysenbach G. Improving the quality of Web surveys: the Checklist for Reporting Results of Internet E-Surveys (CHERRIES). J Med Internet Res. 2004 Sep 29;6(3):e34. doi: 10.2196/jmir.6.3.e34. Erratum in: doi:10.2196/jmir.2042. PMID: 15471760; PMCID: PMC1550605.
